# Conserved and Unique Roles of Chaperone-Dependent E3 Ubiquitin Ligase CHIP in Plants

**DOI:** 10.3389/fpls.2021.699756

**Published:** 2021-07-09

**Authors:** Yan Zhang, Gengshou Xia, Qianggen Zhu

**Affiliations:** Department of Landscape and Horticulture, Ecology College, Lishui University, Lishui, China

**Keywords:** protein quality control, CHIP ubiquitin E3 ligase, ubiquitination, molecular chaperones, heat shock proteins, protein degradation, plant stress responses, chloroplasts

## Abstract

Protein quality control (PQC) is essential for maintaining cellular homeostasis by reducing protein misfolding and aggregation. Major PQC mechanisms include protein refolding assisted by molecular chaperones and the degradation of misfolded and aggregated proteins using the proteasome and autophagy. A C-terminus of heat shock protein (Hsp) 70-interacting protein [carboxy-terminal Hsp70-interacting protein (CHIP)] is a chaperone-dependent and U-box-containing E3 ligase. CHIP is a key molecule in PQC by recognizing misfolded proteins through its interacting chaperones and targeting their degradation. CHIP also ubiquitinates native proteins and plays a regulatory role in other cellular processes, including signaling, development, DNA repair, immunity, and aging in metazoans. As a highly conserved ubiquitin ligase, plant CHIP plays an important role in response to a broad spectrum of biotic and abiotic stresses. CHIP protects chloroplasts by coordinating chloroplast PQC both outside and inside the important photosynthetic organelle of plant cells. CHIP also modulates the activity of protein phosphatase 2A (PP2A), a crucial component in a network of plant signaling, including abscisic acid (ABA) signaling. In this review, we discuss the structure, cofactors, activities, and biological function of CHIP with an emphasis on both its conserved and unique roles in PQC, stress responses, and signaling in plants.

## Introduction

A cell is presented with a continuous stream of misfolded proteins as a result of the stochastic fluctuations, and the presence of destabilizing mutations, stress, and other pathological conditions such as cancers and aging (Hartl and Hayer-Hartl, 2009). These misfolded proteins can jeopardize cell viability due to their non-specific interactions with other cellular components and have a tendency to aggregate to form toxic protein inclusions (Dobson, 2003). An elaborate network of protein quality control (PQC) involving molecular chaperones and protein degradation factors and pathways continually monitors, refolds, and degrades misfolded proteins to maintain the integrity of the proteome (Chen et al., 2011). The ability to constantly replenish and adjust a constituent protein pool is not only a vital component for the modulation of responses under stress conditions but also a steady-state feature under normal conditions to maintain cellular homeostasis (Chen et al., 2011). In addition, the regulated degradation of native proteins in signaling or other necessities is important to execute regulatory programs in response to specific cellular and environmental cues (Varshavsky, 2005, 2017).

All aspects of cellular protein homeostasis rely on molecular chaperones, which promote protein folding, translocation across membrane, and refolding of misfolded or stress-denatured substrates (Hartl et al., 2011; Tittelmeier et al., 2020). Molecular chaperones also play a critical role in preventing the aggregation and targeting of the degradation of misfolded proteins. In eukaryotic cells, these are chaperones associated with protein synthesis by assisting folding of newly synthesized proteins (Albanese et al., 2006), heat shock proteins (Hsps), which are generally classified based on their molecular masses (e.g., Hsp100, Hsp90, Hsp70, Hsp60, and Hsp40), and small heat shock proteins (sHsps) (Hartl et al., 2011). Molecular chaperones such as Hsp70 and Hsp90 have an ATPase activity and can specifically recognize partially folded or misfolded proteins to promote their folding or other conformational changes in an ATP-dependent manner. Molecular co-chaperones interact with molecular chaperones to directly influence their ATPase activity and assist them in protein folding and other functions. Hsp40 proteins are co-chaperones and essential partners of Hsp70 function with a highly conserved region called the J-domain that can activate the ATPase activity of Hsp70 (Chen et al., 2011; Hartl et al., 2011). sHsps usually also function as molecular chaperones that typically associate with and hold misfolded proteins in a reversible state through dynamic but ATP-independent oligomerization state changes, which help to facilitate the refolding or degradation of client proteins by other chaperones such as Hsp70 (Chen et al., 2011). Therefore, different chaperones functionally cooperate with each other, co-chaperones, and other cofactors to promote protein folding, refolding, and degradation.

The ubiquitin-proteasome system (UPS) is a major eukaryotic proteolytic pathway responsible for the clearance of soluble misfolded proteins. Misfolded proteins are usually ubiquitinated by a tightly controlled E1/E2/E3 ubiquitination cascade and targeted for degradation by the 26S proteasome (Pickart and Cohen, 2004). Different linkages of polyubiquitin chains can direct proteins for degradation (i.e., K48) or determine a specific subcellular localization (i.e., K63) (Xu et al., 2008). Different ubiquitin states (mono- vs. polyubiquitination) can also regulate the activity and trafficking of proteins (Akutsu et al., 2016). Misfolded and aggregated proteins can also be degraded by autophagy in the lysosome or vacuole (Yin et al., 2016; Klionsky, 2020). The broad roles of autophagy are primarily mediated by a selective clearance of specifically targeted components through selective autophagy receptors (Gatica et al., 2018). The selective autophagic degradation of protein aggregates or aggrephagy relies on structurally related NBR1 and p62 cargo receptors (Hyttinen et al., 2014; Tan and Wong, 2017). Although the proteasome and autophagy pathways are operated independently with different sets of players, there are reciprocal cross talks between the two degradation pathways in multiple layers (Varshavsky, 2017). Protein ubiquitination and deubiquitination play important roles in controlling the initiation, execution, and termination of autophagy (Kirkin et al., 2009; Kraft et al., 2010; Rogov and Kirkin, 2014; Khaminets et al., 2016).

The mechanisms by which misfolded proteins are recognized by the E3 ubiquitin ligases for ubiquitination are critical for their targeted degradation by either the proteasome or autophagy. Some E3 ligases have been shown to ubiquitinate misfolded proteins in an Hsp70-dependent manner. In the endoplasmic reticulum- (ER-) associated degradation (ERAD), yeast ER membrane-localized E3 ubiquitin ligases Doa10 and Hrd1 can mediate the ubiquitination of misfolded ER and cytosolic substrates in a Hsp70-dependent manner (Burr et al., 2013; Doroudgar et al., 2015; Habeck et al., 2015). Other E3 ubiquitin ligases that recognize misfolded proteins with the assistance of HSP70 include E6AP and MGRN1 proteins (Mishra et al., 2009; Chhangani and Mishra, 2013). In metazoans, a highly conserved E3 ligase carboxy-terminal Hsp70-interacting protein (CHIP) is bound directly to Hsp70 and Hsp90 and ubiquitinates Hsp70/Hsp90-bound proteins for degradation. CHIP can also ubiquitinate proteins with non-canonical ubiquitin chain linkages (e.g., K27 and K63) for regulatory purposes other than proteasomal degradation (Alberti et al., 2002). As such, CHIP has been extensively investigated for its physiological functions and has been established to associate with the development and pathological disorders such as cancers, neurodegenerative diseases, inflammation, and metabolic bone diseases in metazoans (Imai et al., 2002; Sahara et al., 2005; Shin Y. et al., 2005; Dickey et al., 2006; Min et al., 2008; Tetzlaff et al., 2008; Naito et al., 2010; Stankowski et al., 2011; Le et al., 2012; Wang et al., 2012; Sarkar et al., 2014). As a highly conserved ubiquitin ligase, CHIP in plants was first reported almost 20 years ago (Yan et al., 2003) and has been now established to play a critical role in response to a broad spectrum of biotic and abiotic stresses (Zhou et al., 2014; Copeland et al., 2016; Zhang et al., 2021). Other studies have shown that CHIP protects chloroplasts and photosynthesis by coordinating chloroplast PQC both outside and inside the important photosynthetic organelle of plant cells (Shen et al., 2007a,b; Lee et al., 2009; Zhang et al., 2021). CHIP also interacts with and modulates the activity of protein phosphatase 2A (PP2A), a crucial component in a network of plant signaling, including abscisic acid (ABA) signaling (Luo et al., 2006). These results indicate that CHIP is also a key player in PQC in plant cells and plays a broad role in plant stress responses. In this review, we will summarize our current knowledge about the structure, cofactors, activities, and biological function of the CHIP ubiquitin E3 ligase with a particular emphasis on the role of CHIP in stress responses, PQC, and signaling in plants.

## Structures, Cofactors, and Activities of CHIP

**FIGURE 1:**
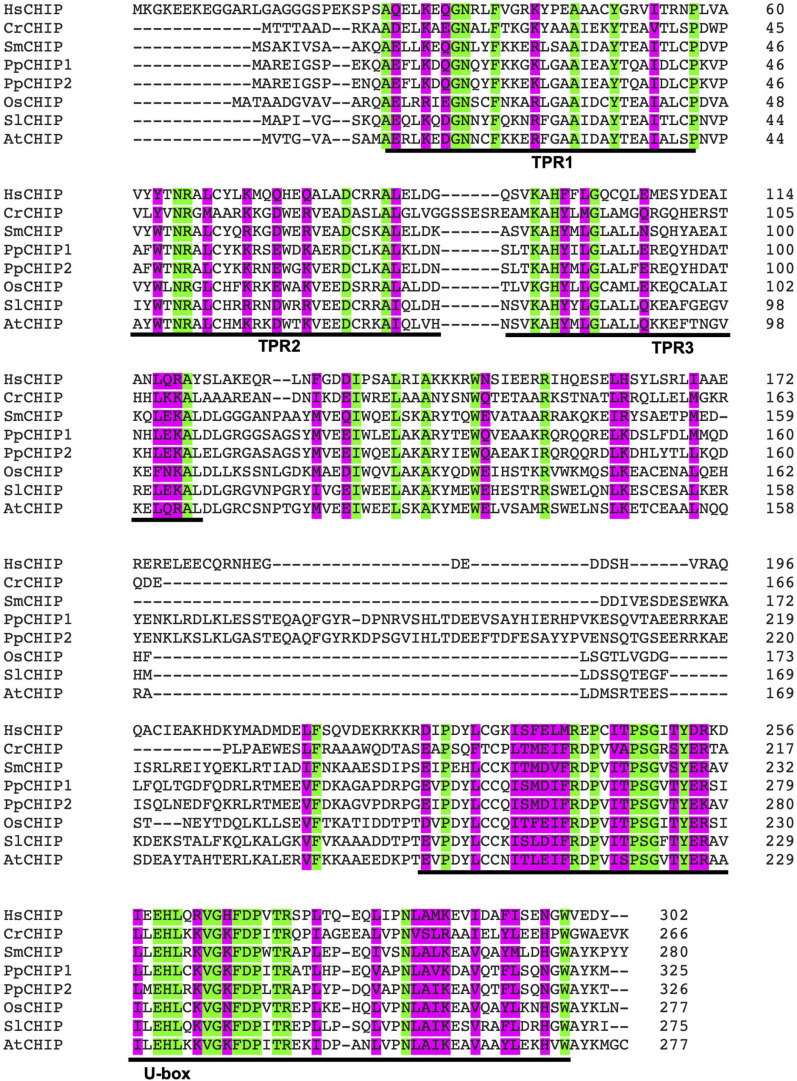
Amino acid sequence alignment of carboxy-terminal Hsp70-interacting protein (CHIP) ubiquitin E3 ligase proteins from human and plants. The accession numbers of the CHIP proteins used in the alignment are: HsCHIP from *Homo sapiens* (AAD33400); CrCHIP from *Chlamydomonas reinhardtii* (Cre.11g479650.t1.2); SmCHIP from *Selaginella moellendorffii* (Selaginella74711); PpCHIP1 and PpCHIP2 from *Physcomitrium patens* (Pp3c14_2850V3.7 and Pp3c10_2570V3.5, respectively); OsCHIP from *Oryza sativa* (LOC_Os05g01460.1); SlCHIP from *Solanum lycopersicum* (Solyc06g083150.2.1); and AtCHIP from *Arabidopsis thaliana* (AT3G07370.1). Identical amino acid residues are indicated in green. Highly similar amino acid residues are indicated in pink. The N-terminal tetratricopeptide repeat (TPR) motifs and the C-terminal B-box domain are also indicated.

**FIGURE 2:**
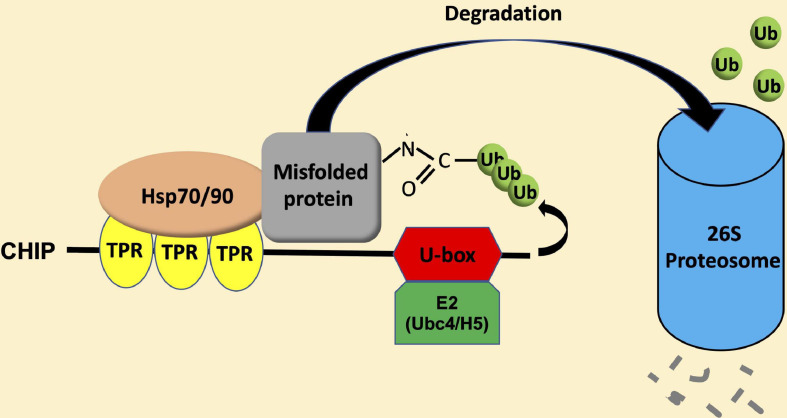
CHIP-mediated ubiquitination and degradation of misfolded proteins. Heat shock protein (Hsp) 70 and Hsp90 recognize misfolded proteins and can switch their role as molecular chaperones from promoting protein folding/maturation to facilitate protein degradation upon binding to the N-terminal TPR domain of CHIP. Upon Hsp70 or Hsp90, CHIP recruits E2 enzymes of the Ubc4/H5 family to ubiquitinate misfolded proteins, and targets their degradation by the 26S proteasome system.

Carboxy-terminal Hsp70-interacting protein is a protein dimer containing an N-terminal tetratricopeptide repeat (TPR) and a C-terminal U-box domain (Zhang M. H. et al., 2005; Page et al., 2015). As shown in Figure 1, there is a substantial amino acid sequence similarity in the TPR and U-box domains among human and plant CHIP proteins. The TPR domain of CHIP is bound directly to a highly conserved EEVD motif located in the C-termini of heat shock cognate (Hsc)/Hsp70 and Hsp90 (Ballinger et al., 1999). The C-terminal U-box domain of CHIP contains E3 ubiquitin ligase activity and polyubiquitin chain extension activity (Jiang et al., 2001). U-Box domains are structurally similar to really interesting new gene (RING) domains but contain two sets of hydrogen-bonding networks in positions of the stabilizing zinc ions in RING domains (Aravind and Koonin, 2000). As depicted in Figure 2, the presence of both chaperone binding and ubiquitin ligase activity suggests a role of CHIP in cellular PQC through the recognition of misfolded proteins by CHIP-bound chaperones and targeting of their ubiquitination and degradation by the 26S proteasome.

Heat shock protein 70 and Hsp90 recognize different types of misfolded proteins (Genest et al., 2019). Hsp70 usually binds short exposed sequences of extended hydrophobic amino acids of newly synthesized protein chains, intermediates of protein translocation, and misfolded proteins. On the other hand, most known substrates of Hsp90 are believed to resemble late-stage folding intermediates in near-native conformations. Therefore, Hsp70-CHIP complexes may target loosely folded proteins while CHIP–Hsp90 complexes might act on more compact misfolded proteins. CHIP binds Hsp70 at the level approximately 10 fold higher than that of Hsp90 and at a much higher affinity (Kundrat and Regan, 2010; Assimon et al., 2015). Furthermore, misfolded proteins were preferentially ubiquitinated through the CHIP-Hsp70 over the CHIP-Hsp90 system (Stankiewicz et al., 2010). Thus, the CHIP-Hsp70 system is the major complex system and mediates the degradation pathway. In this process, there is a switch in the role of Hsp70 and Hsp90 molecular chaperones from promoting protein folding/maturation to facilitate proteasome-mediated degradation *via* lysine48-linked polyubiquitination (Cyr et al., 2002; Wiederkehr et al., 2002). This is apparently achieved through the action of CHIP as a co-chaperone in addition to its activity as an ubiquitin E3 ligase. Hsp70- and Hsp90-mediated protein folding/maturation relies on the coordinated action of a number of co-chaperones such as Hsp40, which load client proteins onto and stimulate the intrinsic ATPase activity of the molecular chaperones. CHIP does not appear to inhibit the ability of Hsp40 to load proteins onto Hsp70, but inhibits the Hsp40-stimulated ATPase activity of Hsp70 (Hohfeld et al., 2001; McDonough and Patterson, 2003). CHIP also inhibits the interaction of Hsp90 with its co-chaperone p23, which promotes the ATP-dependent release of substrates from the polypeptide-binding domain of Hsp90 (Edkins, 2015). These results indicate that, being a co-chaperone and an E3 ubiquitin ligase, CHIP inhibits the chaperone-mediated folding and promote the degradation of substrates by decreasing the ATPase activity of both Hsp70 and Hsp90 and blocking the forward reaction of the Hsp70 substrate-binding cycle and ATP-dependent release from Hsp90 (Edkins, 2015). Upon binding of Hsp70 or Hsp90, CHIP recruits E2 enzymes of the Ubc4/UbcH5 family to ubiquitinate Hsp70 or Hsp90-bound misfolded proteins (Xu et al., 2008; Figure 2). Both the TRP and helical domains play an important role in positioning the U-box domains in the context of a CHIP-chaperone heterocomplex (Zhang et al., 2015).

The identification of the first plant CHIP ubiquitin E3 ligase from the model plant *Arabidopsis thaliana* was reported in 2003 (Yan et al., 2003). Like its orthologs in animals, *Arabidopsis* CHIP contains three TPRs at an N-terminal side and a U-box domain at a C-terminal side. CHIP protein sequences from plants and animals share a strong homology within the TPRs and U-box domains (Yan et al., 2003; Figure 1). Similar to its animal orthologs, *Arabidopsis* CHIP interacts with the ubiquitin-conjugating enzyme (UBC or E2) that belongs to the stress-inducible UBC4/5 class in yeast (Luo et al., 2006). In an *in vitro* assay, *Arabidopsis* CHIP can make ubiquitin polymers very efficiently in the presence of ubiquitin, E1, and E2 of animal sources, indicating that it is an E3 ubiquitin ligase (Yan et al., 2003). Furthermore, coimmunoprecipitation assays showed an interaction of *Arabidopsis* CHIP with the conserved C-terminal CHIP-interacting motif of Hsc70-4 (Lee et al., 2009). These results support a highly conserved nature of both the structure, cofactors, and activities of CHIP proteins from different eukaryotes.

## Broad and Complex Roles of CHIP in Metazoans

Since its discovery in 1999 (Ballinger et al., 1999), the importance of CHIP was revealed by the observation that 20% of the CHIP knockout (CHIP−/−) mice die in the embryonic stages as a result of marked thymic atrophy from reduced ability to cope with stress, and 100% of the CHIP−/− mice die from thermal stress due to the induction of apoptosis in multiple organs (Zhang C. L. et al., 2005). CHIP is encoded by STIP1 homology and U-box containing gene 1 (STUB1) in humans, and their mutations are associated with multiple pathological disorders, including the Gordon Holmes syndrome, brain diseases (e.g., intracranial aneurysm), and lung inflammation. These mutations indicate a critical role of this E3 ubiquitin ligase protein in many important cellular processes and developmental stages in metazoans (Wang et al., 2020).

Carboxy-terminal Hsp70-interacting protein has a broad range of substrates in metazoans. Through coordination with different E2 UBC, CHIP can catalyze K48- (Ubc4/5 E2-) linked polyubiquitination to target the substrate proteins for proteasomal degradation, or K63-(Ubc13-Uev1a E2-) or K27- (unknown E2) linked ubiquitination, which is usually involved in the signaling transduction. CHIP is primarily localized in the cytoplasm but is also found in the nucleus for functions, including the activation of heat shock factor 1 (HSF1) (Dai et al., 2003; Huang et al., 2016). The cytoplasmic CHIP targets not only cytoplasmic proteins but also proteins from the ER, mitochondria [e.g., leucine-rich repeat kinase 2 (LRRK2, and the Golgi complex (e.g., microtubule-associated tau and β-amyloid precursor protein or β-APP) (Joshi et al., 2016). Therefore, the function of CHIP regulates a variety of substrates from different cellular locations and compartments. While the critical role of CHIP in degrading misfolded proteins under normal and stress conditions has been well recognized, CHIP also plays an important role in maintaining the optimal levels of proteins involved in stress responses. For example, CHIP not only functionally regulates Hsp70 as a co-chaperone but also ubiquitinates the molecular chaperone and targets its degradation by a proteasomal pathway (Qian et al., 2006). CHIP preferentially ubiquitinates chaperone-bound substrates, but can also target the degradation of Hsp70 and Hsp90 by the UPS when the misfolded substrates have been depleted (Qian et al., 2006). The sequential targeting of the CHIP-associated chaperone adaptor and bound substrates helps to maintain chaperones at appropriate levels to reflect the status of protein folding in the cytoplasm. CHIP and Hsp90 determine the levels of such proteins as an aryl hydrocarbon receptor (AhR) and SUMO/sentrin protease3 (SENP3), which plays an important role in the cellular response to mild oxidative stress (Morales and Perdew, 2007; Yan et al., 2010). CHIP also helps to maintain the optimal levels of base excision repair enzymes, including DNA polymerase b, DNA ligase III, and x-ray repair cross-complementing group-1 (XRCC1) (Parsons et al., 2008; Sobol, 2008). All these studies show the protective role of CHIP under different stress conditions such as heat shock, DNA damage, and oxidative stress. In the remaining part of this section, we will briefly summarize the roles of CHIP in immunity, neurodegeneration, and aging in metazoan species.

Carboxy-terminal Hsp70-interacting protein mediates the ubiquitination of a wide variety of protein substrates with critical roles in regulating various aspects of innate and adaptive immunity (Zhan et al., 2017). These substrates include: (1) Nuclear factor kappa B (NF-κB) inducing kinase (NIK), an essential Ser/Thr kinase and a central factor in the non-canonical NF-κB signaling pathway and in the development and function of the immune system in both mice and humans (Yin et al., 2001). CHIP dimers act as scaffold proteins and interact with both Hsp70-NIK and tumor necrosis factor receptor-associated factor 3 (TRAF3) *via* the CHIP-TPR domain. While CHIP and TRAF3 individually cause a modest reduction of NIK protein levels, the combination of both CHIP and TRAF3 promotes a much greater NIK degradation (Jiang et al., 2015). (2) Toll-like receptors (TLRs) play an important role in the recognition of pathogen-associated molecular patterns (PAMPs) (Kawai and Akira, 2011). TLRs can be divided into two subgroups according to cellular localization and PAMP ligands. CHIP can direct the Hsp70-mediated assembly of some TLR complexes and the ubiquitination of associated components in a K63-linked manner, leading to an activation of the signaling pathway (Yang et al., 2011). On the other hand, CHIP mediates the inhibitory effect of Hsp70 on TLR4 signaling through the ubiquitination and degradation of TLR4 (Afrazi et al., 2012). (3) Nitric oxide synthase (NOS) generates NO from the amino acid L-arginine. CHIP downregulates both neuronal and inducible NOSs through the ubiquitination and proteasome-dependent degradation (Peng et al., 2004; Chen et al., 2009). (4) Retinoic acid-inducible gene-I (RIG-I)-like receptors (RLRs) play an essential role in innate immunity by initiating inflammatory responses, such as the production of proinflammatory cytokines and type I interferons, upon the detection of replicating viruses particularly during the early stages of viral infection in the cytoplasm (Kato et al., 2011; Ramos and Gale, 2011). RIG-I is the most important factor that initiates RLR signaling by RNA viruses. CHIP mediates K48-linked ubiquitination and degradation of RIG-I. By interacting directly with CHIP, the transcription factor signal transducer and activator of transcription 4 (STAT4) can stabilize RIG-I to block its association with CHIP in the cytoplasm of RNA virus-challenged macrophages (Zhao et al., 2016). CHIP also plays role in several immune-related diseases, including systemic lupus erythematosus and airway inflammation in chronic respiratory diseases (Zhan et al., 2017).

Neurodegenerative diseases are characterized by a massive loss of specific neurons (Joshi et al., 2016). The accumulation of aberrant proteins and protein aggregate is associated with the pathology of the most frequent neurodegenerative diseases, including Alzheimer’s disease, amyotrophic lateral sclerosis (ALS), and Parkinson’s disease. The main cause of Alzheimer’s disease is the formation of intracellular neurofibrillary tangles of a phosphorylated form of microtubule-associated tau protein (Binder et al., 2005). In action with the UbcH5B E2 enzyme, CHIP-Hsp70 complex polyubiquitinates the phosphorylated form of tau *via* both K48 and K62 linkages and targets it for proteasomal or autophagic degradation (Petrucelli et al., 2004). CHIP in conjugation with Hsp70 and Hsp90 can also reduce the accumulation of toxic amyloid beta Aβ42 peptide, which can lead to neuronal loss of function or synaptic loss; loss of neurons may lead to death in later stages (Kumar et al., 2007). ALS occurs due to mutations in the antioxidant enzyme superoxide dismutase 1 (SOD1) located on mitochondrial membranes and transactive response DNA-binding protein-43 (TDP-43) (Joshi et al., 2016). CHIP mediates Hsc/Hsp70-dependent polyubiquitination and degradation of SOD1 *via* 26S proteasome (Urushitani et al., 2004; Choi and Lee, 2010). The B-cell lymphoma 2- (BCL2-) associated athanogene 3 (Bag3)/HspB8/Hsc70/CHIP complex also inserts mutant SOD1 protein into autophagosome and targets their degradation by autophagy (Crippa et al., 2010). Parkinson’s disease also begins with the formation of proteotoxic inclusions from Pael-R (Parkin-associated endotheliun receptor-like receptor, a G-protein-coupled receptor), α-synuclein, and LRRK2 inside the neurons (Ko et al., 2009). Pael-R can accumulate in the ER and cause ER stress, leading to neuronal death. CHIP mediates the dissociation of Hsp70 and the Parkin E3 ligase and increases the ubiquitination of Pael-R and subsequent degradation by the proteasome (Imai et al., 2002). The cytoplasmic fibrillar aggregates of α-synuclein known as Lewy bodies are a hallmark of Parkinson’s disease. CHIP directs interaction with α-synuclein and targets its degradation (Shin Y. G. et al., 2005). A mutation in mitochondria-associated LRRK2 protein is involved in the onset of familial Parkinson’s disease and may also be a substrate of CHIP (Ding and Goldberg, 2009).

Carboxy-terminal Hsp70-interacting protein is involved in the regulation of aging by affecting the activity and stability of such proteins as the sirtuin family of proteins play important roles in DNA repair and genome integrity (Ronnebaum et al., 2013). Aging is associated with a progressive decline in protein homeostasis (proteostasis), which promotes the risk for protein aggregation diseases. As a key regulator of proteostasis, CHIP deficiency increased levels of the insulin receptor (INSR), and reduced worms and flies lifespan (Tawo et al., 2017). The membrane-bound INSR defines metabolism and aging by regulating the insulin and insulin-like growth factor 1 (IGF1) signaling (IIS) pathway. CHIP directly mediates monoubiquitylation and endocytic-lysosomal turnover of INSR to promote longevity. During aging and under conditions of proteotoxic stress, however, CHIP is recruited for the degradation of misfolded proteins, thereby reducing its engagement with the degradation of the INSR (Tawo et al., 2017). These results support that CHIP-mediated proteolysis plays an important role in the competitive relationship between proteostasis and longevity regulation.

## Role of CHIP in Plant Tolerance to Extreme Temperatures

As sessile organisms, plants are inevitably exposed to a broad spectrum of adverse environmental conditions such as high or cold temperature, drought, and soil salinity. These harmful conditions cause damage to cellular structures and molecules including proteins (Gong, 2021). For example, heat stress under high temperature causes protein misfolding and aggregation. A large number of studies have shown that ubiquitination and protein degradation play critical roles in plant responses to various abiotic stresses. Stress conditions such as high temperature induce the expression of multiple polyubiquitin genes in plants (Christensen et al., 1992; Genschik et al., 1992; Sun and Callis, 1997) and the overexpression of a monoubiquitin gene increased a tolerance to multiple stresses in transgenic plants (Guo et al., 2008). Autophagy is a major protein degradation pathway in eukaryotes and also plays an important role in plant tolerance to multiple abiotic stresses. Extensive studies have established ubiquitination, which mediates the recognition of protein aggregates and other damaged constituents by selective autophagy under stress condition (Zhang and Chen, 2020; Luo et al., 2021). In plants, the selective autophagy receptor NBR1 contains an ubiquitin-association domain and plays a critical role in plant stress tolerance by targeting autophagic degradation of ubiquitinated protein aggregates under stress conditions (Zhou et al., 2013, 2014). Furthermore, gene mutations for the 19S regulatory particle subunits of the 26S proteasome compromise plant tolerance to salt, UV radiation, and heat shock (Wang et al., 2009), further supporting that UPS plays a critical role in general plant stress responses. However, a majority of these reported ubiquitin E3 ligases regulate plant stress responses through the modulation of the levels of regulatory proteins such as transcription factors (Mandal et al., 2018). Chaperone-dependent CHIP ubiquitin E3 ligase is among only a few ubiquitin E3 ligases that have been analyzed for their direct roles in degrading misfolded and unwanted proteins that accumulate under stress conditions.

*Arabidopsis* CHIP is a single-copy gene, and its expression is upregulated by several stress conditions such as cold, heat, and salt (Yan et al., 2003). As discussed earlier, *Arabidopsis* CHIP proteins have the same TPR and U-box domains and the E3 ligase activity as metazoan CHIP proteins. However, increased CHIP expression is not associated with increased stress tolerance; in fact, CHIP overexpression in transgenic *Arabidopsis* plants compromises a tolerance to both cold and heat stress (Yan et al., 2003). The phenotypes of the CHIP-overexpressing transgenic plants are unexpected given the established roles of the E3 ubiquitin ligase in the removal of misfolded and damaged proteins. However, chronic overexpression of CHIP can also alter essential signaling pathways and can lead to deleterious effects in human cells (Stankowski et al., 2011). To address the role of CHIP using a loss-of-function approach, we have previously isolated two independent transfer DNA (T-DNA) insertion mutants for *Arabidopsis* CHIP. Both *chip* mutants grow and develop normally and display no detectable morphological phenotypes when grown under normal conditions (Zhou et al., 2014). However, the mutations of *Arabidopsis* CHIP increase sensitivity to a variety of abiotic stresses, including high temperature, salt, and oxidative stress. The *chip* mutants are also altered in sensitivity to exogenous ABA (Zhou et al., 2014). Recently, we have also analyzed the role of the tomato CHIP gene in heat tolerance (Zhang et al., 2021). Like *Arabidopsis* CHIP gene, tomato CHIP is induced by heat treatment. Virus-induced silencing of the tomato CHIP gene led to increased heat sensitivity based on the development of an increase in heat stress symptoms, reduced photosynthesis, and increased ion leakage under high temperature (Zhang et al., 2021). These results from both the mutations and silencing of the CHIP genes from *Arabidopsis* and tomato conclusively demonstrate the important function of the ubiquitin E3 ligase in response to extreme temperatures (Table 1).

**TABLE 1:** Roles of carboxy-terminal Hsp70-interacting protein (CHIP) E3 ubiquitin ligase in plants.

Biological process	Target protein	Function	References
Temperature stress responses	Misfolded proteins	Degradation of misfolded proteins	Yan et al., 2003; Zhou et al., 2014; Zhang et al., 2021
Plant immunity	Unknown	Positive regulation of basal defense, influenced by temperature	Copeland et al., 2016
Chloroplast PQC	Chloroplast precursor proteins	Degradation of misfolded and unimported chloroplast proteins in the cytosol	Lee et al., 2016
	Clp and FtsH subunits	Maintaining of homeostasis of ClpP and FtsH subunits	Shen et al., 2007a,b,c,d
ABA signaling	PP2A	Stimulation of PP2A activity and, in turn, ABA-mediated and other PP2A-regulated stress response	Luo et al., 2006

Several approaches have been taken to determine whether the critical role of CHIP in plant heat tolerance is mediated through its action in the targeting of misfolded or stress-damaged proteins for degradation by either the proteasome or autophagy. Indeed, reduced heat tolerance of *Arabidopsis chip* mutants was associated with an increase in the accumulation of protein aggregates under high temperature (Zhou et al., 2014). Likewise, tomato plants with CHIP gene silencing accumulate more protein aggregates than the control tomato plants under high temperature (Zhang et al., 2021). These phenotypes of reduced heat tolerance and the accumulation of heat-induced protein aggregates under heat stress are strikingly similar to those of the mutants for the selective autophagy receptor NBR1 (Zhou et al., 2013, 2014) and, therefore, an effort was also taken to determine their functional relationship through an analysis of the *chip/nbr1* double mutants. When compared with the chip and nbr1 single mutants, the *chip/nbr1* double mutant plants are further compromised in heat tolerance and in the removal of heat-induced protein aggregates (Zhou et al., 2014). Furthermore, we observed high ubiquitination of insoluble protein aggregates accumulated under high temperature in the *chip* single and *chip/nbr1* double mutant plants and concluded that E3 ubiqutin ligases other than CHIP are required for the ubiquitination of heat-induced protein aggregates in NBR1-mediated selective autophagy (Zhou et al., 2014). These results strongly suggest that NBR1 and CHIP mediate two separate but complementary PQC pathways during plant heat stress responses. Proteomic profiling of heat-induced protein aggregates in the *chip* and *nbr1* single and *chip/nbr1* double mutants supports this interpretation (Zhou et al., 2014). In the relatively early stage of heat stress, aggregates from a substantial number of proteins differentially accumulate in the *nbr1* and *chip* mutants. After 6 h of heat stress, the levels of protein aggregates for a substantial number of proteins, including catalases and Rubisco activase, were three to five times higher in the *nbr1* mutant than in the *chip* mutant. Catalases and Rubisco activase are highly heat sensitive and prone to form aggregates at high temperature (Chen et al., 1993a,b; Salvucci et al., 2001). Other proteins, including light-harvesting complex (LHC) subunits, preferentially accumulates as aggregates in the *chip* mutant during the early stage of heat stress. Previous studies have shown that CHIP recognizes LHC subunits and acts with HSC70-4 in the specific degradation of the plastid-destined LHC subunit proteins in a plastid import mutant (Lee et al., 2009). These findings suggest a potential determinant for the selection of protein substrates by the two pathways: proteins such as Rubisco activase and catalases that are highly aggregate-prone are preferentially cleared by selective autophagy, probably because those aggregates are difficult to unfold to pass through a small 13 Å wide central cavity of the 20S proteolytic core (Nandi et al., 2006). CHIP-mediated proteasome system, on the other hand, can effectively degrade misfolded but still soluble proteins such as the cytosolic precursors of LHC proteins as they can apparently be unfolded. After more extended heat stress, the total levels of protein aggregates increased in both the *chip* and *nbr1* mutants but their differential accumulation became less clear (Zhou et al., 2014). Furthermore, the levels of aggregates for most of the detected proteins were higher in the *chip/nbr1* double mutant than in the *chip* and *nbr1* single mutants, supporting that CHIP- and NBR1-mediated pathways are complementary in the degradation of heat-induced misfolded proteins (Zhou et al., 2014).

## Role of CHIP in Plant Immunity

Plants have sophisticated immune systems for the recognition of and defense against diverse microbial pathogens. Pattern-recognition receptors on the cell surface can recognize PAMPs to trigger PAMP-triggered immunity (PTI), which is effective at preventing infection by many microbes (Dangl et al., 2013). Successful pathogens are able to suppress PTI and promote virulence by delivering effector molecules into the plant cells (Dangl et al., 2013). Some of the effectors may be recognized by intracellular resistance (R) protein to trigger rapid and robust effector-triggered immunity (ETI). Most R proteins from plants contain nucleotide-binding, leucine-rich repeat domains (NLRs) with Toll-interleukin-1 receptor (TIR), or coiled-coil (CC) domains at their N termini (Jones and Dangl, 2006). The activation of ETI is often associated with hypersensitive cell death (Jones and Dangl, 2006), and therefore, ETI signaling must be tightly regulated in healthy plants to prevent spontaneous cell death or other deleterious effects on plant growth (Li et al., 2001; Yang et al., 2010).

Some NLRs and other regulators of plant defense responses are regulated posttranslationally through degradation by the proteasome system (Cheng et al., 2011; Huang et al., 2014), indicating roles of E3 ubiquitin ligases in plant immunity. Indeed, T-DNA knockout alleles of *Arabidopsis* CHIP gene exhibit enhanced disease susceptibility to the oomycete pathogen *Hyaloperonospora arabidopsidis* (*H.a.*) Noco2. The mutants also show slightly enhanced susceptibility to the virulent bacterial pathogen Pseudomonas *syringae* pv. *maculicola* (*P.s.m*.) ES4326 but respond normally to *P. syringae* pv. *tomato* (*P.s.t*.) DC3000 strains carrying either *AvrRpt2* or *AvrRps4* (Copeland et al., 2016). SA accumulation is also normal in the mutants after the infection with a high dose of *P.s.t.* DC3000 *AvrRps4*. Transgenic *Arabidopsis* plants overexpressing CHIP are also normal in response to the virulent bacterial pathogen under normal growth conditions. Interestingly, when plants were grown at 20°C, and transferred to18°C for at least 1 week before infection, there is a significant reduction in bacterial growth following *P.s.m.* ES4326 inoculation (Copeland et al., 2016). Thus, the results from both the knockout mutants and overexpression lines support a positive role of CHIP in plant basal resistance, which also appears to be influenced by temperature (Table 1).

Carboxy-terminal Hsp70-interacting protein functions with but also targets molecular chaperone Hsp90, which plays important role in the regulation of R protein stability and function (Kadota and Shirasu, 2012). A number of co-chaperones of Hsp90, including suppressor of G2 allele of S-phase kinase-associated protein 1 (SGT1) and required for Mla12 resistance 1 (RAR1), also play important roles in plant immunity. Furthermore, a point mutation in a suppressor of *npr1*, constitutive 1 (SNC1), an *Arabidopsis* TNL protein, leads to enhanced resistance to virulent pathogens by the autoimmune *snc1* mutant associated with dwarfism and curled-leaf morphology (Li et al., 2001). Therefore, the role of CHIP in plant immunity could be attributed to its action in the regulation of the levels of HSP90 and specific TNL proteins such as SNC1. However, western blotting indicated that the mutations of *Arabidopsis CHIP* gene did not affect the accumulation of HSP90 or mutant SNC1 proteins (Copeland et al., 2016). It is possible that *Arabidopsis* CHIP targets an unknown negative regulator of immunity, which would accumulate in the *chip* plants to promote disease susceptibility (Copeland et al., 2016). It is also plausible that the loss of *CHIP* function causes the accumulation of abnormal proteins, thereby contributing to a cellular environment that reduces immune signaling and basal resistance to virulent pathogens (Copeland et al., 2016).

## Role of CHIP in Chloroplast PQC

**FIGURE 3:**
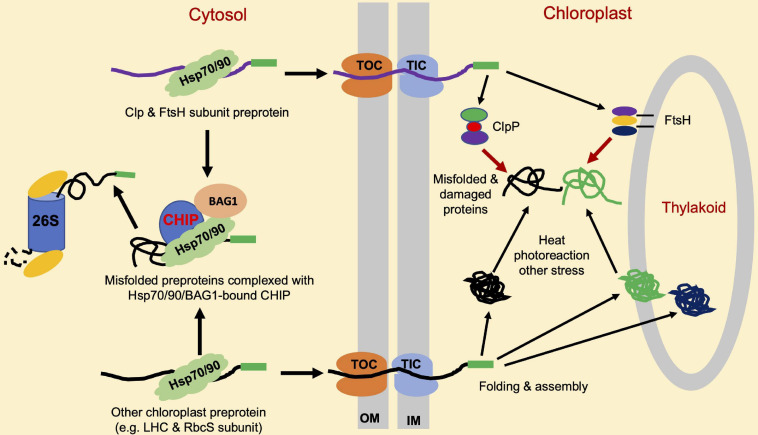
Roles of CHIP in chloroplast protein quality control (PQC). A majority of chloroplast proteins are nuclear-encoded and synthesized as precursor proteins (pre-proteins) in the cytosol. Import of these precursors proteins into chloroplasts is through the joint action of molecular machines named translocon at the outer envelope membrane (OM) of chloroplast (TOC), and translocon at the inner envelope membrane (OIM) of chloroplast (TIC). CHIP targets the degradation of misfolded and accumulated chloroplast precursor proteins such as light-harvesting complex (LHC) and Rubisco small (RbcS) subunits, which can aggregate and interfere with the chloroplast protein import. CHIP can also target the degradation of accumulated and misfolded ClpP and filamentation temperature sensitive H (FtsH) protease subunit pre-proteins to help maintain the balance among different subunits for assembly into active protease complexes. ClpP and FtsH target the degradation of misfolded, physiologically, and stress-damaged proteins insides the chloroplasts.

Like mitochondria, chloroplasts are a semiautonomous organelle with an endosymbiotic origin. Chloroplasts contain 2,500–3,000 proteins, more than 95% of which are encoded by nuclear genes and are synthesized at cytosolic ribosomes as chloroplast precursor proteins and transported as unfolded proteins into the stroma through the translocon complexes in the outer chloroplast (TOC) and inner chloroplast (TIC) envelope membranes (Sun et al., 2021). Sufficiently low levels of chloroplast precursor proteins in the cytosol are necessary for avoiding the saturation of the import machinery, which can inhibit efficient translocation. The formation of non-specific protein aggregates tends to occur from the chloroplast precursor proteins that accumulate in the cytosol in plant cells (Lee et al., 2009). In *Arabidopsis* plastid protein import2 mutant plants, which has a severe defect in protein import into chloroplasts, the genes for the cytosolic Hsc70-4, CHIP E3 ligase and BAG1 co-chaperone are highly induced. Hsc70-4 recognizes specific sequence motifs in transit peptides and acts with CHIP to promote pre-protein degradation by the proteasome system. BAG proteins function as nucleotide exchange factors for Hsc70 and play important roles in protein homeostasis in mammalian cells (Briknarova et al., 2001). *Arabidopsis* BAG1 plays a crucial role in Hsc70-4/CHIP-mediated proteasomal clearance of misfolded and unimported plastid proteins in the cytosol (Lee et al., 2016). These results indicate that CHIP plays a crucial role in chloroplast PQC (Table 1). As depicted in Figure 3, the role of CHIP in chloroplast PQC is mediated mainly through the action of CHIP by targeting the degradation of plastid-destined precursors to prevent their accumulation in the cytosol (Figure 3). The induction of Hsc70-4 and CHIP in the *Arabidopsis* mutant impaired in plastid protein import (Lee et al., 2009) suggests the production of retrograde signals from chloroplasts, which are transmitted to the nucleus to regulate the level of cytosolic pre-proteins. Plants with lower Hsc70 levels are abnormal in embryogenesis and contain high levels of reactive oxygen species (ROS) and a monoubiquitinated Lhcb4 precursor in seedlings (Lee et al., 2009). Chloroplast ROS is known to rapidly activate general control non-derepressible 2 (GCN2) kinase, which can phosphorylate eukaryotic translation initiation factor 2a (eIF2a) to suppress the synthesis of chloroplast proteins precursors in the cytosol (Lokdarshi et al., 2020a,b).

Upon arrival at chloroplast stroma, pre-proteins undergo proteolytic processing for a cleavage of presequences and are further folded, assembled, or sorted for translocation by molecular chaperones to intrachloroplast compartments, including thylakoids (McKinnon et al., 2020). Inside the chloroplasts, misfolded, physiologically, and stress-damaged proteins are degraded by proteases. There are more than 20 organelle proteases in plants (Majsec et al., 2017), including the ubiquitous AAA + family of ATPase caseinolytic proteases (Clps), filamentation temperature-sensitive H (FtsH), long-filament phenotype (LON) protease, and degradation (DEG) periplasmic proteases (van Wijk, 2015). These proteases remove both imported and plastid-encoded substrates. For instance, photodamaged photosystem II (PSII) reaction center proteins such as D1 and D2 are primarily degraded by the thylakoid membrane-localized FtsH protease FtsH2 (VAR2) (Kato et al., 2009; Dogra et al., 2019). ATP-dependent Clp proteases are responsible for protein degradation in chloroplasts and mitochondria (Moreno et al., 2018). Interestingly, CHIP also appears to control PQC inside the chloroplasts indirectly through the regulation of chloroplast proteases. *Arabidopsis* CHIP interacts with a proteolytic subunit, ClpP4, of the chloroplast Clp protease *in vivo*, and ubiquitylates ClpP4 *in vitro* (Shen et al., 2007b). CHIP can also interact with ClpP3 and ClpP5 in yeast and ubiquitylate the two subunits *in vitro* (Wei et al., 2015). CHIP-overexpressing reduced the steady-state level of ClpP4 in plants under high-intensity light conditions, suggesting that CHIP targets ClpP4 for degradation to regulate the Clp proteolytic activity in chloroplasts under certain stress conditions. *Arabidopsis* CHIP also interacts with FtsH1, a subunit of the chloroplast FtsH protease complex (Shen et al., 2007a). AtCHIP can ubiquitinate FtsH1 *in vitro*, and CHIP-overexpressing plants contain a reduced steady-state level of FtsH1 under high-intensity light conditions, suggesting that the ubiquitylation of FtsH1 by CHIP also leads to the degradation of FtsH1 *in vivo*. In addition, in CHIP-overexpressing plants, the steady-state level of FtsH2, another subunit of the chloroplast FtsH protease complex, is also reduced under high-intensity light conditions. CHIP also interacts with FtsH2 *in vivo*, suggesting that FtsH2 is also a protein substrate for CHIP in plant cells (Shen et al., 2007a). In CHIP-overexpressing plants, the D1 PSII reaction center protein, a substrate of FtsH protease, is not efficiently removed by FtsH under high-intensity light conditions, supporting that FtsH subunits are substrates of CHIP *in vivo* and their protein levels are reduced due to CHIP overexpression in chloroplasts. CHIP interacts with cytosolic Hsp70 and the precursors of FtsH1 and FtsH2 in the cytosol, and Hsp70 also interacts with FtsH1. These protein-protein interactions appear to be enhanced under high-intensity light conditions, suggesting that Hsp70 might be partly responsible for increased degradation of FtsH1 and FtsH2, in CHIP-overexpressing plants. Therefore, CHIP acts in coordination with bound molecular chaperones and plays an important role in PQC in chloroplasts (Figure 3 and Table 1).

The fact that CHIP targeting chloroplast proteases important for chloroplast PQC is counterintuitive given the established cytoprotective role of CHIP. There could be multiple reasons for the seemingly contradictory findings on the role of CHIP in cellular PQC. First, even though CHIP plays a critical role in protecting the integrity of the proteome, and its level and activity require tight control to avoid non-specific degradation of native proteins (e.g., Hsp70), particularly in the absence or presence of low levels of non-native protein substrates. This interpretation is consistent with the observation that both mutations and overexpression of CHIP lead to compromised tolerance to extreme temperature (Yan et al., 2003; Zhou et al., 2014). Second, both ClpP and FtsH complexes are heteromeric AAA-ATPase complexes composed of related ClpP and FtsH subunits, respectively (Nishimura et al., 2016). Proper stoichiometry of different ClpP and FtsH subunits in their respective protease complexes could be important for their stability or activity. This is consistent with the observations that both antisense and overexpression of ClpP4, one of the ClpP core subunits, cause chlorotic phenotypes in *Arabidopsis* (Wei et al., 2015). Importantly, the overexpression of CHIP rescues the chlorotic phenotypes of both ClpP4 antisense and overexpressing plants. The unbalanced levels of Clp core proteins in ClpP4 antisense and overexpressing plants with the overexpression of CHIP were similar to wild-type levels (Wei et al., 2015), suggesting that CHIP regulates ClpP core proteins. *Arabidopsis* CHIP also interacts with ClpP3 and ClpP5 and ubiquitinates the two ClpP subunits *in vitro* (Wei et al., 2015). Based on these results, it is likely that, through the selective degradation of ClpP and FtsH subunits, CHIP positively regulates the homeostasis of ClpP and FtsH proteolytic subunits to protect chloroplast functions (Figure 3 and Table 1). Similar results have been obtained from transgenic tobacco plants, supporting the conserved nature of the regulation of the chloroplast protease by CHIP (Wei et al., 2015).

As discussed earlier, CHIP plays a critical role in heat tolerance in both *Arabidopsis* and tomato. Compromised heat tolerance in *Arabidopsis chip* mutants was associated with the reduction of photosynthesis and the accumulation of insoluble protein aggregates of chloroplast photosynthetic proteins (Zhang et al., 2021). Therefore, CHIP could play a similar role in chloroplast PQC to promote plant heat tolerance. At high temperature, the capacity of a protein precursor import into chloroplasts is known to decrease (Dutta et al., 2009), leading to their increased accumulation in the cytosol. Heat stress could also cause increased levels of misfolded chloroplast precursor proteins. These unfolded or misfolded chloroplast precursor proteins would be recognized by molecular chaperones Hsp70 and Hsp90 and ubiquitinated by chaperone-dependent CHIP for degradation by the proteasome. Interestingly, CHIP interacts with the Rubisco small (RbcS) and Lhcb6 in yeast cells (Luo et al., 2006), both of which are found to accumulate in the *chip* mutants under high temperature (Zhang et al., 2021). RbcS and Lhcb proteins are among the most abundant nuclear-encoded chloroplast proteins and their direct recognition by CHIP underscores a particularly important role of the E3 ligase in chloroplast PQC. Furthermore, heat stress could lead to increased misfolding of ClpP and FtsH protease subunit precursors. Heat stress could also lead to the disruption of the proper stoichiometry of the protease precursors in the cytosol due to their differential stability under high temperature. Chaperone-dependent CHIP E3 ligase could positively regulate the homeostasis of Clp proteolytic subunits and maximize the production of functional chloroplasts under heat stress. FtsH proteases are involved in the degradation of not only unassembled thylakoid membrane proteins (Malnoe et al., 2014) and photodamaged PSII D1 protein (Kato and Sakamoto, 2014) but also heat-denatured proteins in chloroplasts (Adam et al., 2019). Like *Arabidopsis chip* mutants, loss-of-function *Arabidopsis* FtsH11 mutants have reduced photosynthetic capacity and are highly susceptible to elevated temperatures (Chen et al., 2018).

## Role of CHIP in ABA Signaling

**FIGURE 4:**
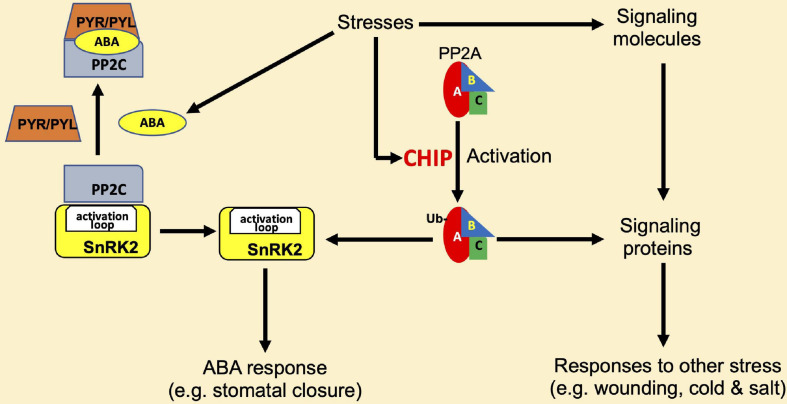
Regulation of protein phosphatase 2A (PP2A) by CHIP in plant stress responses. CHIP interacts with and monoubiquitinates the A1 and A3 subunits of PP2A to stimulate its activity. PP2A has regulatory roles in abscisic acid (ABA) signaling and affects ABA responses such as stomatal closure through an association with the sucrose non-fermenting-1-related protein kinase 2- (SnRK2-) type kinases. SnRK2 is inactivated by the interacting 2C-type protein phosphatases (PP2C), and this action of PP2C is inhibited by PYR/PVL ABA receptors in the presence of ABA. PP2A also regulates signaling of other phytohormones such as jasmonic acid and ethylene and affects responses to other stresses such as wounding, cold, and salt stresses.

In metazoans, CHIP proteins not only promote the degradation of Hsp70 and Hsp90 substrate proteins through the proteasome system but also play roles in the modulation of activity and stability of proteins in signaling and other regulatory processes (Joshi et al., 2016). Plant CHIP proteins also play a regulatory role in signal transduction. *Arabidopsis* CHIP interacts with the A3 subunit of PP2A (Luo et al., 2006). The PP2A family of protein phosphatases in eukaryotes are heterotrimeric complexes, which comprise a catalytic subunit C, a regulatory subunit B, and a scaffolding subunit A (Durian et al., 2016). The *Arabidopsis* genome contains 5 different genes encoding C subunits, 3 genes for the A subunits, and 17 genes encoding the variable regulatory B subunits (Janssens and Goris, 2001; Uhrig et al., 2013; Durian et al., 2016). These subunit isoforms differentially assemble into multiple PP2A complexes that regulate plant growth, development, metabolism, and stress responses (Uhrig et al., 2013). CHIP can monoubiquitinate not only A3 but also A1 subunit of PP2A *in vitro* (Luo et al., 2006). The scaffold subunit A1 of PP2A is also known as ROOTS CURL IN NAPHTHYLPHTHALAMIC ACID1 (RCN1), which functions as a positive regulator in ABA-related pathways (Kwak et al., 2002), and *rcn1* mutants also display pleiotropic phenotypes in other phytohormone signaling pathways (Deruere et al., 1999; Larsen and Cancel, 2003; Muday et al., 2006; Blakeslee et al., 2008). Unlike other CHIP-interacting proteins and substrates, the overexpression of *Arabidopsis* CHIP does not reduce the protein levels of PP2A subunits under normal growth conditions (Luo et al., 2006). In fact, the activity of PP2A is increased in CHIP-overexpressing plants in the dark or under low-temperature conditions (Luo et al., 2006). Thus, as shown in Figure 4, unlike with other CHIP target proteins, the ubiquitination of PP2A A subunits by CHIP does not lead to their degradation, rather it increases the PP2A activity under certain conditions.

The functions of PP2A have been linked to the regulation of plant signaling and responses to a variety of biotic and abiotic stresses (Figure 4). The tomato subunits LePP2Ac1 and LePP2Ac2 and the potato PP2A catalytic subunit StPP2Ac2 play roles in wounding-induced jasmonic acid signaling (Pais et al., 2009) while the tomato subunits LePP2Ac1, LePP2Ac2, and LePP2Ac3 function in cold stress (Pais et al., 2009). Salinity stress inducing genes-encoding rice PP2A catalytic subunit OsPP2A-1-5 and the potato subunits StPP2Ac1, StPP2Ac2a, StPP2Ac2b, and StPP2Ac3 (Yu et al., 2003, 2005; Pais et al., 2009), and wheat TaPP2Ac-1 acts as a positive regulator of salt stress responses (Xu et al., 2007). In addition, by associating with the sucrose non-fermenting-1-related protein kinase 2- (SnRK2-) type kinases, PP2A participates in ABA-mediated stomatal closure (Waadt et al., 2015; Figure 4). Unlike the *rcn1* mutant, which has reduced PP2A activity due to a mutation in the A1 subunit gene of PP2A, CHIP-overexpressing plants display an increased sensitivity to ABA treatment (Luo et al., 2006). Since PP2A is involved in low-temperature responses in plants (Yan et al., 2003), the low-temperature sensitivity of CHIP-overexpressing plants might be in part due to the change in PP2A activity. These results strongly suggest that CHIP may function upstream of PP2A in stress-responsive signal transduction pathways under certain stress conditions such as low temperature (Figure 4 and Table 1).

## Conclusion and Future Perspective

Eukaryotic cells produce a continuous stream of misfolded proteins particularly under stress conditions, which need to be refolded or degraded to maintain the cellular protein homeostasis and integrity of the proteome. Evolutionarily conserved CHIP ubiquitin E3 ligase selectively ubiquitinates the target proteins by recognizing the non-native state in a molecular chaperone-assisted manner. In metazoans, CHIP has been extensively analyzed for its roles not only in cellular PQC but also in signaling and other regulatory processes associated with a variety of pathological disorders, including cancers, neurodegenerative diseases, immunity, and aging. Since its first report in 2003, plant CHIP proteins from *Arabidopsis* and tomato have been analyzed for their roles in plant responses to biotic and abiotic stresses. Plant CHIP proteins appear to play a particularly important role in the control of chloroplast protein quality. In coordination with Hsc70-4, CHIP mediates the degradation of chloroplast protein precursors accumulated in the cytosol. CHIP may also indirectly control the protein quality inside the chloroplasts by regulating the levels and balance of chloroplast ClpP and FtsH protease complex subunits. CHIP also enhances the activity of PP2A, a crucial component in a network of plant signaling, including ABA signaling, that is closely linked with plant stress responses. These findings demonstrate that plant CHIP proteins have conserved a biological function in cellular PQC by targeting the degradation of misfolded and other damaged non-native proteins to maintain protein homeostasis. It is also apparent that plant CHIP proteins have also evolved to regulate the stability and activity of proteins involved in biological processes, such as photosynthesis and ABA signaling, that are unique to plants. When compared to the extensive analysis and knowledge about CHIP in metazoans, our understanding of plant CHIP proteins is still very limited. There have been a relatively very few number of reported studies on the functional characterization of plant CHIP proteins, through an analysis of the *chip* mutants and overexpression lines in responses to pathogens and abiotic stresses. Almost all these reported studies on plant CHIP have been using *Arabidopsis*, and the knowledge about the ubiquitin E3 ligase in other plant species is almost completely lacking. Unlike in metazoan species, the number of identified substrates of plant CHIP proteins is still very small. Even for those identified plant CHIP substrates, it is still not completely clear how they are targeted for ubquitinated in terms of the types of polyubiquitin chain linkages (K48, K63, or K27) and the ubiquitin states (mono- vs. polyubiquitination), which often affect the stability, activity, subcellular trafficking, and localization. In addition to Hsc/Hsp70 and Hsp90, CHIP acts in conjugation with other specific factors, including co-chaperones and E2 ubiquitin E2 conjugating enzymes, and this information for plant CHIP proteins is also missing. Over the past two decades, a variety of molecular and biochemical tools and approaches have been developed for the identification and quantification of ubiquitin-modified substrates as well as approaches to quantify the length, abundance, linkage type, and architecture of different ubiquitin chains. These tools can be employed in combination with genetic and multi-omics approaches to decipher the broad and complex roles of CHIP in plants as well established in metazoan species. A better knowledge about the function, action mechanisms, and regulation of a highly conserved CHIP ubiquitin E3 ligase will provide important new insights into the molecular basis of plant growth and fitness under both normal and stress conditions, which can be exploited to improve crop plants.

## Author Contributions

YZ and QZ conceived the idea. YZ wrote the manuscript. GX evaluated the manuscript. All authors contributed to the article and approved the submitted version.

## Conflict of Interest

The authors declare that the research was conducted in the absence of any commercial or financial relationships that could be construed as a potential conflict of interest.
